# Mechanistic Insights into the Anti-Inflammatory and Anti-Proliferative Effects of Selected Medicinal Plants in Endometriosis

**DOI:** 10.3390/ijms262210947

**Published:** 2025-11-12

**Authors:** Oliwia Burdan, Natalia Picheta, Julia Piekarz, Karolina Daniłowska, Filip Gajewski, Krzysztof Kułak, Rafał Tarkowski

**Affiliations:** 1 Students Scientific Association—I Chair and Department of Gynaecological Oncology and Gynaecology, Medical University of Lublin, Staszica 16 Str., 20-081 Lublin, Poland; natalia.picheta2812@gmail.com (N.P.); karolina02812@gmail.com (K.D.);; 2 I Chair and Department of Gynaecological Oncology and Gynaecology, Medical University of Lublin, Staszica 16 Str., 20-081 Lublin, Poland

**Keywords:** *Curcuma longa*, endometriosis, *Glycyrrhiza glabra*, phytotherapy, *Silybum marianum*, *Zingiber officinale*

## Abstract

Endometriosis involves oestrogen-dependent chronic inflammation and the abnormal proliferation of ectopic endometrial tissue. Conventional hormonal therapies suppress systemic oestrogen, but do not fully address local oxidative and inflammatory signalling. This review provides a mechanistic synthesis of recent molecular evidence. This evidence is on four FDA-recognized (Food and Drug Administration) medicinal plants. These are *Curcuma longa*, *Zingiber officinale*, *Glycyrrhiza glabra*, and *Silybum marianum*. The review highlights their capacity to modulate key intracellular pathways. These pathways are implicated in endometriosis. The review covers the integration of phytochemical-specific actions within NF-κB- (nuclear factor kappa-light-chain-enhancer of activated B cells), COX-2-(Cyclooxygenase-2), PI3K/Akt-(PI3K/Akt signaling pathway), Nrf2/ARE-(Nuclear factor erythroid 2–related factor 2) and ERβ-(Estrogen receptor beta) mediated networks, which jointly regulate cytokine secretion, apoptosis, angiogenesis and redox balance in endometrial lesions. Curcumin downregulates COX-2 and aromatase while activating Nrf2 signalling, shogaol from ginger suppresses prostaglandin synthesis and induces caspase-dependent apoptosis, isoliquiritigenin from liquorice inhibits HMGB1-TLR4–NF-κB (High Mobility Group Box 1, Toll-like receptor 4) activation, and silymarin from milk thistle reduces IL-6 (Interleukin-6) and miR-155 (microRNA-155) expression while enhancing antioxidant capacity. Together, these phytochemicals demonstrate pharmacodynamic complementarity with hormonal agents by targeting local inflammatory and oxidative circuits rather than systemic endocrine axes. This mechanistic framework supports the rational integration of phytotherapy into endometriosis management and identifies redox-inflammatory signalling nodes as future translational targets.

## 1. Introduction

Endometriosis is a multifactorial, estrogen-dependent, chronic inflammatory disorder characterized by the ectopic implantation of endometrial tissue outside the uterine cavity [1]. The pathological manifestations vary considerably, from superficial peritoneal deposits and ovarian endometriomas to deeply infiltrating lesions involving the intestines, bladder, and even extra-pelvic sites such as the lungs and skin [2]. Affecting approximately 10% of women of reproductive age, it represents one of the most prevalent causes of chronic pelvic pain and infertility worldwide [3]. The disease significantly impairs patients’ quality of life, often manifesting as dysmenorrhea, dyspareunia, and chronic fatigue, and has been associated with comorbid inflammatory and autoimmune disorders including rheumatoid arthritis, psoriasis, and migraine [4,5,6].

Current diagnostic protocols rely on a combination of clinical evaluation, imaging, and surgical confirmation. Although ultrasound and MRI (Magnetic Resonance Imaging) assist in lesion localization, laparoscopy followed by histopathological verification remains the diagnostic gold standard [7,8]. Disease severity is routinely graded according to the revised American Society for Reproductive Medicine (rASRM) classification, which assesses the extent of adhesions and implants [9].

Conventional management strategies target pain control, suppression of ectopic endometrial proliferation, and prevention of recurrence. Pharmacological treatment typically includes nonsteroidal anti-inflammatory drugs (NSAIDs) and hormonal agents such as gonadotropin-releasing hormone (GnRH) analogues, progestogens, and combined oral contraceptives [2,10,11]. While these interventions reduce hormonal activity and alleviate symptoms, they are frequently limited by relapse after discontinuation, side effects, and contraindications in women seeking pregnancy. Surgical excision of lesions via laparoscopy remains an option for refractory cases, but recurrence rates remain high. Therefore, multidisciplinary approaches incorporating physiotherapy, diet modification, and psychological support have become integral to comprehensive care [11,12].

Given the chronic inflammatory and oxidative nature of endometriosis, there is growing scientific interest in complementary strategies that modulate these molecular pathways [13]. Phytotherapy represents one such promising adjunctive approach. Bioactive plant-derived compounds, such as polyphenols and flavonoids, have demonstrated the ability to suppress pro-inflammatory cytokines, inhibit angiogenesis, and restore redox balance—mechanisms directly implicated in endometriotic lesion progression [14]. Integrating phytochemicals with standard hormonal therapy may thus offer a multimodal therapeutic paradigm capable of addressing both the endocrine and inflammatory components of the disease.

## 2. Pathomechanism and Development of Endometriosis

In order to help patients suffering from endometriosis and select appropriate treatment, it is essential to understand the condition’s pathogenesis. Many hypotheses have been proposed to explain the development of endometriosis, and they all essentially boil down to a dysregulated hormonal balance and increased levels of pro-inflammatory cytokines [12]. The retrograde menstruation hypothesis, first proposed in 1927, has attracted the most support. During menstruation, endometrial tissue moves into the pelvic cavity through the fallopian tubes. This tissue reaches the peritoneal cavity, where it proliferates in the mesothelial cells, and then invades pelvic structures [15]. Interestingly, endometrial reflux occurs in 90% of women and is a physiological event. However, researchers conjecture that the difference between physiological and pathological processes depends on the histological and hormonal composition of the reflux, which has not yet been precisely specified. It is presumed that prostaglandins (PGE) play a role in increased reflux in women with endometriosis by disorganising uterine muscle contraction. Prostaglandins are synthesised in the peritoneal cavity by ectopic endometrial cells, and the enzyme responsible for their synthesis is cyclooxygenase (COX) [16]. A particularly important isoform is COX-2, which is overexpressed in endometriosis. The most important prostaglandin (PGE) in the development of endometriosis is PGE-2, as it stimulates oestrogen synthesis in ectopic foci of the endometrium by mediating the transcription of the aromatase gene (P450arom). Additionally, PGE-2 enhances aromatase expression through steroidogenic factor-1 (SF1), which binds to the promoter of the aromatase gene (P450arom) [17]. This factor is pathologically overexpressed in ectopic endometrial cells. These processes lead to increased oestrogen levels, which are involved in a feedback loop that increases PGE-2 synthesis [18].

High levels of COX-2 and, consequently, PGE-2 have been linked to a reduced tendency for endometrial cells to undergo apoptosis, as well as increased cellular proliferation, angiogenesis, and the pain associated with endometriosis [19]. Some scholars have disagreed with the theory of retrograde menstruation. Sampson justified his dissatisfaction with the reflux theory on the grounds that it does not explain the abnormal localisation of endometriosis foci in locations extraperitoneal and far removed from the pelvic area [20]. He therefore proposed that endometriosis translocation is related to its transport through blood or lymphatic vessels. This theory is supported by clinical evidence due to the presence of endometrium in uterine blood vessels and congestion in sentinel lymph nodes. Additionally, the coelomic metaplasia and Müllerian remnants hypothesis states that lesions of endometrial origin arise in situ from embryonic remnants. According to this theory, endometriosis is the result of the abnormal migration and differentiation of embryonic cell remnants originating from Müllerian ducts at an early stage of organogenesis. This theory is pertinent in that it seems to explain the occurrence of endometriosis in teenage girls before or shortly after their first menstrual period [12]. Recently, epigenetic theory has been gaining popularity. Epigenetic modifications involve dynamic and reversible changes within chromatin that alter gene expression. The most significant of these are hypo- or hypermethylation, histone modification, and microRNA production [21].

Hypermethylation of the *HOXA10* (Homeobox A10) protein gene is the most well-described phenomenon. Its presence has been demonstrated in both mouse models and women with ectopic endometriosis, as opposed to healthy women [22]. Furthermore, the invasiveness of endometriosis cells is regulated by hypermethylation. In endometriosis, Let-7 microRNA is hypermethylated, which leads to decreased expression of this microRNA and inhibits *KRAS* expression, promoting the growth and invasiveness of ectopic endometriosis foci [23].

The role of histones in endometriosis is not fully understood. Studies have shown significant hypoacetylation of histones in eutopic and ectopic tissue lining cells in diseased women compared to healthy women. The acetylation levels of histones H3 and H4 are also lower in eutopic and ectopic tissue in women with the disease [24].

In addition to the established theories, understanding the pathophysiology of pain development during endometriosis is also important. Pain is a common and troublesome symptom reported by patients. Endometriosis is characterised by elevated levels of pro-inflammatory factors such as interleukin 6 and 8 (IL-6/IL-8), tumour necrosis factor alpha (TNF-α), and prostaglandin E2 (PGE2). The cyclic release of pain and inflammatory mediators activates visceral and peritoneal fibres, thus increasing pain sensitivity [25].

An important initial aspect of managing endometriosis is pain management. As well as conventional medications such as NSAIDs, natural methods can be employed to support pain management, such as herbal remedies. Turmeric, ginger, milk thistle and liquorice root have anti-inflammatory properties and can help to regulate oestrogen levels and reduce oxidative stress. This makes them potential agents that can combat endometriosis pain and perhaps even reduce ectopic endometriosis foci [25].

**Figure 1:**
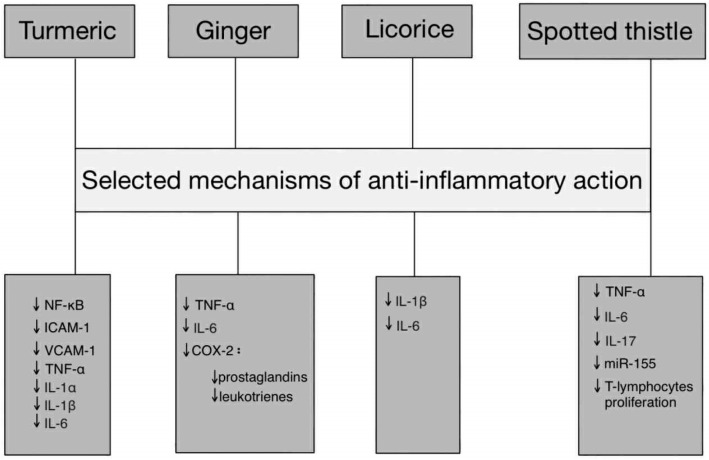
Pathway-based representation of anti-inflammatory actions of Turmeric, Ginger, Licorice, and Spotted thistle. Abbreviations: TNF-α—tumor necrosis factor alpha, IL—interleukin, NF-κB—nuclear factor kappa-light-chain-enhancer of activated B cells, ICAM-1—intercellular adhesion molecule 1, VCAM-1—vascular cell adhesion molecule 1, miR-155—microRNA-155, COX-2—cyclooxygenase, ↓—downregulation.

Treatment for endometriosis must involve interventions that can modulate the multidimensional nature of the disease, which involves inflammatory (IL-6, TNF-α, CCL2), hormonal (ERβ, aromatase, 17β-estradiol) and oxidative (MDA, ROS, SOD/GPx) factors. Plant compounds such as polyphenols and flavonoids have been found to exhibit various properties. These include anti-inflammatory, antioxidant (ROS neutralisation and SOD/GPx elevation) and oestrogen system-modulating properties (e.g., aromatase inhibition and oestrogen receptor modulation). Preliminary preclinical studies and a diet high in antioxidants have demonstrated reductions in oxidative stress markers and improvements in the cytokine profile in women with endometriosis [26]. Therefore, phytotherapeutic interventions may be a valuable addition to standard treatment strategies, particularly given the chronic nature of the disease and the limitations of hormone therapy, not to mention the need for long-term control of inflammation and oxidative stress (Figure 1).

## 3. Methodology

This literature review was conducted by searching scientific articles from 2015 to 2025. The bases were the PubMed, Scopus, and Google Scholar databases, using the following keywords: *Curcuma longa*; Endometriosis; *Glycyrrhiza glabra*; Phytotherapy; *Silybum marianum*; *Zingiber officinale*. Among the 2148 articles in the available database, 80 publications were included in this study after detailed analysis.

The inclusion criteria were: topics related to endometriosis, the pathomechanisms of the disease, or the use of phytotherapy in its treatment, full access to the text, and the presence of a DOI identifier.

The review excluded unreviewed publications, those not available in full text or without a DOI number, as well as works of low methodological quality and works in languages other than English. Studies not related to endometriosis and duplicates of previous results were also excluded.

## 4. Turmeric (*Curcuma longa*)

### 4.1. Mechanism of Action and Pharmacologically Active Substances

*Curcuma longa*, commonly known as turmeric, belongs to the ginger family and is widely cultivated and used in South-West Asian countries [27]. The plant contains several substances that have therapeutic effects, including curcumin (60–70%), demethoxycurcumin (20–30%), and bisdemethoxycurcumin (10–15%) [28]. These substances are attributed anti-inflammatory, anti-cancer, antimicrobial, anti-diabetic, anti-ageing and antioxidant effects. Thanks to these properties, the herb can be used in complementary therapies for inflammatory diseases, such as intestinal and joint diseases, cancer, and chronic pain [29]. The Food and Agriculture Organization/World Health Organization (FAO/WHO) and the European Food Safety Authority (EFSA) have approved a daily dose of 0–3 mg/kg. Additionally, the US Food and Drug Administration (FDA) has approved the herb as a botanical [30]. The FDA has stated that the daily dose of curcumin should be between 4 and 8 g/day [31].

### 4.2. Preclinical Trail Data

Turmeric is a herbal remedy that can be used to treat many diseases, including endometriosis. It exerts strong anti-inflammatory, anti-proliferative, anti-angiogenic and pro-apoptotic effects on cells in pathological endometrial tissue. Given the vast array of mechanisms affected by turmeric’s active substances, it is the subject of numerous studies on human, animal (mainly mouse) and in vitro models, investigating its potential as a therapeutic agent in endometriosis treatment [32]. By comprehensively inhibiting the processes of endometriosis cell proliferation, angiogenesis and inflammation, curcumin counteracts the progression of the pathology that reduces the quality of life of endometriosis patients, alleviating the symptoms of the disease. This makes it an effective addition to holistic therapy for this female reproductive system pathology. In an experimental study, endometrial cells were analysed from 23 patients of reproductive age ranging from 24 to 45 years. The study sample size comprised 14 ectopic linings and nine eutopic endometriosis linings. The cells were cultured in the presence of curcumin at two concentrations: 20 µmol/L and 50 µmol/L for 48 h. This made it possible to observe the effect of curcumin on the growth and survival of the cells in the endometriosis sublayers tested. Significant changes in cell proliferation were observed, as confirmed by the MTT assay: both ectopic and eutopic cells showed reduced growth by 40% at 50 µmol/L curcumin. In addition, cell cycle analysis revealed a notable rise in the proportion of cells in the G1 phase and a decrease in the S phase, particularly in the group exposed to a higher dosage of curcumin. This suggests an inhibition of DNA synthesis in endometrial tissue cells. Turmeric was also found to significantly decrease vascular endothelial growth factor (VEGF) expression, indicating a potential mechanism for its anti-angiogenic effect. Applying turmeric at a concentration of 50 µmol/L also induced apoptosis, with 4.7% of ectopic endometrial cells exhibiting early apoptosis and 28.4% exhibiting late apoptosis after two days of testing. These results suggest that curcumin may be a promising therapeutic agent for the treatment of endometriosis. Turmeric has potent anti-inflammatory properties, making it an effective agent in therapies related to the pathological activation of chemokines and cytokines. Thanks to these properties, turmeric is gaining increasing recognition in natural medicine as a means of combatting inflammation. This was demonstrated in a study in which tissue biopsies were taken from the endometrium of six women. The control group consisted of three women without endometriosis, while the study group consisted of three patients with laparoscopically confirmed endometriosis. All biopsy specimens were placed in Dulbecco’s Eagle medium and treated with curcumin at concentrations of 5 and 10 μg/mL for 24 and 48 h. The concentrations of the analysed cytokines and chemokines were determined using specific immunoassays at each tested concentration and time period. The research-relevant cytokines were TNF-α, vascular endothelial growth factor (VEGF), platelet-derived growth factor (PDGF), interferon gamma (IFNγ), fibroblast growth factor (FGF), IL-1β, IL-1α, IL-2, IL-4, IL-5, IL-6, IL-7, IL-8, IL-9, IL-10, IL-12, IL-13, IL-15 and IL-17, as well as the chemokines eotaxin and granulocyte colony-stimulating factor. Curcumin significantly inhibited the secretion of almost all the tested cytokines and chemokines, except for the anti-inflammatory cytokines IL-10 and IL-12, the concentrations of which increased significantly after 48 h in each trial. The highest inhibition of cytokine secretion was observed at a concentration of 10 µg/mL after a 48 h incubation period for the constitutional biopsy specimens. Turmeric may therefore be a safe component of endometriosis therapy [33,34]. Table 1 presents the approximate change in the concentrations of the tested substances after curcumin treatment, following 48 h of observation.

A study by Kim et al. on mouse models showed that curcumin has potent anti-inflammatory effects in the context of endometriosis by inhibiting the NF-κB pathway, which is activated by the central cytokine of the inflammatory process (TNF-α). Consequently, curcumin significantly suppressed the expression of the adhesion proteins ICAM-1 (Intercellular Adhesion Molecule) and VCAM-1 (Vascular Cell Adhesion Molecule), which play a pivotal role in inflammatory responses and cell adhesion. This was confirmed by immunofluorescence microscopy, Western blot analysis, and qRT-PCR (quantitative Reverse Transcription Polymerase Chain Reaction), which showed normalisation of ICAM-1 and VCAM-1 expression at protein and mRNA levels in endometrial lining cells after curcumin treatment. These results demonstrate curcumin’s capacity to regulate surface and cytoplasmic inflammatory markers, thereby mitigating TNF-α-induced inflammatory responses in endometriosis [35]. Numerous studies have confirmed turmeric’s ability to stimulate apoptosis of endometriosis cells. This mechanism involves the inhibition of the phosphoinositide 3-kinase/protein kinase B (PI3K/Akt) pathway, which usually plays an anti-apoptotic role by stimulating the expression of B-cell lymphoma extra-large (Bcl-xL) and B-cell chronic lymphocytic leukaemia/lymphoma 2 (Bcl-2) proteins, as well as caspase-9-inhibitor of apoptosis (XIAP). Curcumin increases the ratio of the pro-apoptotic Bax protein to the anti-apoptotic Bcl-2 protein, releases cytochrome c and activates caspase-9 and caspase-8, thereby initiating apoptosis. Curcumin simultaneously reduces the expression of Bcl-2 and cyclin D1, thereby further inhibiting cell proliferation. In animal models, curcumin has been shown to reduce the weight and volume of endometrial lesions and induce endometriosis cell death in a dose-dependent manner through modulation of the PI3K/Akt and extracellular signal-regulated kinase (ERK) pathways [36,37]. This evidence supports the thesis that turmeric is effective against endometriosis. With its numerous components and unique properties, turmeric may play a key role in improving the lives of patients with endometriosis. Its anti-inflammatory and antioxidant effects, as well as its ability to modulate processes such as apoptosis, angiogenesis and the invasion and adhesion of endometrial lesions, make turmeric a promising adjunct in the treatment of this disease.

While further research is required, the potential of turmeric in dietary prevention and treatment of endometriosis indicates its multidirectional effects on the pathology of the reproductive system. Previous studies suggest that turmeric can alleviate symptoms and slow the progression of the disease, which could greatly improve the quality of life for women with endometriosis. Further clinical research into its mechanisms of action and efficacy is needed to discover its full potential.

### 4.3. Overcoming Pharmacokinetic Limitations of Curcumin

Curcumin has various pharmacokinetic limitations, including low water solubility, chemical instability under physiological conditions, limited penetration of enterocytes, intense phase II metabolism, and rapid elimination from circulation. Following oral administration, conjugated metabolites, notably glucuronides and sulfates, are present. Concurrently, free curcumin concentrations in plasma are found to be negligible. The minimal systemic availability thus makes it challenging to translate in vitro effects to clinical effects. When administered orally to rats at a dose of 2 g/kg, the maximum serum concentration of curcumin was found to be 1.35 ± 0.23 μg/mL after 0.83 h [38].

Increased solubility, stability, and often controlled release are all possible thanks to liposomes and lipid nanocarriers. Preliminary preclinical work and pharmacokinetic studies have demonstrated improved area under the curve (AUC) and better tissue penetration. Lipid nanoparticle (LNP) technology, which is widely used for other drugs, is also being investigated for curcumin. However, standardisation of production and safety assessment are significant challenges for clinical translation [39].

Phospholipid formulations (phytosomes) combine curcuminoids with phosphatidylcholine. This improves amphiphilic distribution. This increases intestinal absorption and tissue concentrations. Pharmacokinetic studies have demonstrated a significant increase in total absorption. This has been demonstrated, for example, by Meriva. For certain curcuminoids, relative AUC has improved by a factor of twelve. Higher tissue concentrations have also been detected. A study conducted by Gary N. Asher et al. found higher concentrations in the rectal mucosa after phytosome administration [40].

## 5. Ginger (*Zingiber officinale*)

### 5.1. Mechanism of Action and Pharmacologically Active Substances

*Zingiber officinale*, commonly known as ginger, belongs to the ginger family and is widely used in China as a spice and in traditional medicine. The plant contains numerous active substances, including gingerol, zingerone, shogaol, paradol and camphene, and has many pharmacological properties, including anti-inflammatory, antimicrobial, antiemetic, analgesic, anticancer and antioxidant effects [41,42]. Since ancient times, it has been used to alleviate cramps, muscle pain, sore throats, indigestion, constipation, fever, infectious diseases, hypertension and dementia [43]. The FDA classifies the plant as ‘generally recognised as safe’ (GRAS), and the optimal dose of the herb is 1000 mg of dried extract [44]. Additionally, ginger reduces areas of pathological endometrial tissue and has demonstrated the capacity to stimulate and support fertility in women with endometriosis.

### 5.2. Preclinical Trial Data

José Meneses de Morais Filho et al. conducted a study in which endometriosis was induced by autotransplantation in Wistar rats. The control group (16 females) was force-fed a 0.9% sodium chloride solution, while the test group (also 16 females) was force-fed 0.5 mg/100 g of fresh *Zingiber officinale* extract. The aforementioned fluids were administered for 14 days. After the rats were euthanized, peritoneal lavage fluid was collected to assess tumour necrosis factor alpha (TNF-α) and interleukin 6 (IL-6), which are the most relevant to the pathogenesis of endometriosis. Pathological endometriotic tissue was also excised and measured. The final mean autotransplant volumes were larger in the control group (120.92 mm^3^ ± 78.91) than in the test group (40.50 mm^3^ ± 19.57) (*p* = 0.01). There was no difference in TNF-α (*p* = 0.51) or IL-6 (*p* = 0.12) levels. The degree of lesion disappearance was higher in the study group (1 ± 0.92) than in the control group (2.25 ± 1.16) (*p* = 0.03). The study concluded that ginger extract reduced and caused the disappearance of autotransplanted endometriosis foci [45]. Women use NSAIDs to relieve pain. NSAIDs provide analgesic effects by inhibiting COX-2 and reducing prostaglandin synthesis. Many women, unaware of the serious side effects of NSAIDs, take them in large quantities, buying these drugs without a prescription. Ginger contains components such as gingerol, shogaol, paradol, zingerone, and gingerdione, which inhibit COX-2, thereby inhibiting prostaglandin and leukotriene synthesis. Additionally, shogaol acts as an agonist of the transient receptor potential vanilloid 1 (TRPV1) channel, which transmits physical and chemical stimuli. Therefore, prolonged exposure to shogaol results in long-term pain relief [46].

In a study involving 16 BALB/c nude mice, female mice aged four to five weeks were injected with Ishikawa cells subcutaneously to create an endometrial tumour model. Sixteen days later, once the tumour volume in each mouse had reached 90 mm^3^, the mice were randomly divided into two groups. One group was treated with 6-shogaol (50 mg/kg) and the other received 0.1% dimethyl sulfoxide (DMSO) as a neutral substance. Treatment lasted for 15 days, with 6-shogaol administered intraperitoneally once daily. Tumour volume was measured, and treatment efficacy was assessed by the T/C ratio (58.12%). The T/C ratio is the mean RTV of the 6-shogaol-treated group divided by the mean RTV of the control group. × 100%. RTV is the quotient of the tumour volume measured every other day and the initial tumour volume. At the end of the experiment, the mice were sacrificed, and histological examinations confirmed a reduction in tumour cell proliferation in the tumours of the mice in the study group. Several mechanisms were observed as a result of 6-shogaol treatment that inhibited tumour growth. Firstly, 6-shogaol was found to inhibit tumour cell proliferation, as confirmed by histological studies. Immunohistochemical staining revealed that nuclei positive for Ki-67 were significantly more prevalent in the 6-shogaol-treated group than in the control group. This resulted in a reduction in the number of dividing cells within the tumour. In addition, 6-shogaol induced changes in the cell cycle, causing tumour cells to arrest in the G1 or G2/M phases and preventing further cell division. Furthermore, 6-shogaol induced apoptosis of tumour cells by activating caspases 3, 7, 8 and 9, thereby inducing programmed cell death and contributing to the reduction in the number of viable tumour cells. Taken together, these findings suggest that 6-shogaol shows therapeutic potential in the treatment of endometriosis by inhibiting growth and inducing apoptosis, both of which are disrupted in this pathology [47].

### 5.3. Cinical Trial Data

In 2016, a single-blind clinical trial was conducted with 200 female medical students aged 18–25 who were suffering from moderate to severe painful menstruation. The participants were randomly divided into four groups, each of which took a different intervention for five days of their menstrual cycle: ginger capsules (500 mg twice daily), vitamin D (1000 IU daily), vitamin E (100 IU daily), or a placebo. All patients were also prescribed the analgesic drug mefenamic acid at a dose of 250 mg twice per day. Statistical analysis showed no significant differences in pain intensity between the groups prior to the study (*p* = 0.52), but after the first and second months of the intervention, significant differences emerged (*p* < 0.001). The greatest reduction in pain was seen in the ginger group, where scores on the visual analogue scale (VAS) decreased by an average of 3.36 in the first month and 3.88 in the second, confirming ginger’s effectiveness as a natural treatment for menstrual pain—one of the more troublesome symptoms experienced by endometriosis patients [48]. Ginger has also been the focus of fertility research, as fertility is impaired in women with endometriosis. In 2019, a study involving 42 white rats was conducted, with the rats being divided into two main groups. Each group had three subgroups, which were given 100 mg, 200 mg or distilled water (control group) of ginger powder, respectively. Group 1 (5-day treatment): Ginger was administered daily for five days, corresponding to one oestrus cycle. Group 2 (10-day treatment): A similar regimen was administered over 10 days, covering two estrous cycles.

At the end of the study, the animals were sacrificed, and their ovaries and endometrium were subjected to a detailed histopathological analysis. Angiogenesis was determined by evaluating VEGF (Vascular Endothelial Growth Factor) expression, while oxidative stress was assessed by the presence of eNOS (endothelial Nitric Oxide Synthase) substances. In the 5-day group, the subgroup that received 100 mg of ginger exhibited significantly higher numbers of antral follicles and VEGF in the ovarian lining (*p* < 0.05) compared to the control group. Similar results were observed in the 10-day group. In the subgroup that received 100 mg of ginger, VEGF in the endometrium and eNOS in the ovarian lining were significantly higher (*p* < 0.05) than in the control group. In both main groups, the subgroups that received 200 mg of ginger showed no statistical difference compared to the control group. Ginger, especially at a dose of 100 mg administered for five days, affects folliculogenesis and angiogenesis, thereby improving ovarian and endometrial health. An increase in the number of antral follicles, as well as higher VEGF and eNOS expression, indicates a positive effect on processes related to ovarian development. This may support the treatment of endometriosis and infertility. Ginger’s therapeutic potential in the context of infertility, particularly in women with endometriosis, may be attributed to its pro-angiogenic properties and its capacity to mitigate oxidative stress [49].

## 6. Licorice (*Glycyrrhiza glabra*)

### 6.1. Mechanism of Action and Pharmacologically Active Substances

*Glycyrrhiza glabra*, commonly known as liquorice, is native to Eurasia and North Africa [50]. It has been used since ancient times to treat asthma, fever, liver disease, neuralgia, and skin and lung diseases [51]. It contains many active substances, including glycyrrhizin, a triterpenoid saponin, and the glycosides liquiritigenin and liquiritin [52]. Consequently, it exhibits a variety of pharmacological properties, including anti-inflammatory, anti-diabetic, anti-ulcer, diastolic, antioxidant and antidepressant properties. Due to these properties, it can be used in adjunctive therapy for various conditions. The herb has been granted GRAS status by the FDA [53,54].

### 6.2. Preclinical Trial Data

In an experiment, the End1/E6E7 human endometriosis cell line was implanted into 18 mice. Two weeks after surgery to verify the adoption and growth of endometrial tissue in the mice’s peritoneum by ultrasound, the study proceeded. The mice were then randomly divided into three groups of six. The control group received corn oil orally, while the other two groups received isoliquiritigenin (ISL) at doses of 1 mg/kg and 5 mg/kg, respectively. After 28 days, the weight and volume of the endometrial lesions were examined. The volumes, dimensions and weights of the lesions in the study groups were evidently smaller than those in the control group. Table 2 presents the results indicating the antiproliferative effect of ISL.

The anti-inflammatory effect of ILS was also analysed. Blood samples were collected from the hearts and endometrial lesions of mice participating in the study. In the ILS-treated group, the levels of IL-1β in the endometrial lesions and of IL-6 in the serum and endometrial lesions were lower than in the control group. These results support the anti-inflammatory theory of ILS use in endometriosis. The expression of apoptosis-regulating proteins in endometrial lesions was examined using Western blot analysis. Expression of the pro-apoptotic proteins Bax, Bcl-2 and caspase-3 was significantly higher than in the control group. Estrogen receptor β expression was also examined. It was significantly inhibited by ILS [55]. Table 3 presents the results indicating the anti-inflammatory effects of ILS.

Another study was performed on a larger group of rats, comparing the effects of liquorice, cyclooxygenase-2 (celecoxib) and a gonadotropin-releasing hormone analogue (Diferelin). Forty-four female rats were each implanted with two pieces of endometrial tissue on their peritoneum. The rats were divided into four equal groups. The control group received 0.5 mL of 0.9% saline orally per day; the second group received 3000 mg/kg/day of licorice root extract orally; the third group received 50 mg/kg of celecoxib twice daily in 0.5 mL of saline orally. The fourth group received a single intramuscular injection containing 3 mg/kg of dipherelin. The rats were administered the test substances for a period of six weeks. After this time, the rats were sacrificed and the endometrial implants were excised during laparotomy. The length, width, and height of each implant were accurately measured using Collis rulers, based on the formula π/6 × length × width × height. All endometrial implants were fixed in formalin, embedded in paraffin wax, cut into 5 µm slices and stained with haematoxylin and eosin. To classify the persistence of epithelial cells in tissue implants, the Keenan scoring system was employed. A score of 0 indicates an absent epithelial layer, while scores of 1, 2 and 3 indicate poorly, moderately and well-preserved epithelial layers, respectively. The percentage of haemoglobin-laden macrophages (HLM) was measured in all samples. In the licorice study group, the mean area and volume values of the endometrial implants were significantly lower than in the control group, with *p* values of 0.042 and <0.001, respectively. The mean area and volume of endometrial implants in the celecoxib group were lower than in the rat control group, but these differences were not statistically significant (*p* = 0.953 and *p* = 0.818, respectively). The mean area and volume of the group receiving diphereline were significantly lower than in the control group (*p* < 0.001 and *p* < 0.001, respectively). Histopathological preparations from tissue implants showed a higher HLM count. This is a marker of chronic bleeding and provides confirmation of the diagnosis of endometriosis. The HLM value remained unchanged following treatment with celecoxib or saline solution. However, the HLM value decreased significantly after treatment with licorice or diphereline. The results of this study support the therapeutic potential of licorice in inhibiting the growth of endometrial implants [57].

### 6.3. Cinical Trial Data

A randomised, triple-blind study was conducted to compare the effects of *Glycyrrhiza glabra* L. and ibuprofen on primary dysmenorrhoea. Sixty women aged 18–25 participated in the study. The participants were randomly divided into two groups. One group received 400 mg of ibuprofen tablets every eight hours and 5 mL of placebo syrup twice daily. The other group received 5 mL of *G. glabra* syrup (150 mg/mL) twice daily and a placebo tablet every eight hours. The women took the test substances from the first day of their period until the fifth day, for two consecutive cycles. They were asked to report the intensity of their pain before taking the first dose and the greatest pain relief experienced up to two hours after taking the last dose. This was measured using the VAS. Ultimately, 26 patients in the *G. glabra* group and 24 patients in the ibuprofen group completed the study. Analysis of the results showed a significant difference in pain reduction before and after treatment in both the G. glabra group (*p* < 0.001) and the ibuprofen control group (*p* < 0.001). However, there was no significant difference in pain relief between the *G. glabra* and ibuprofen groups (*p* = 0.151). Adverse effects of the test substances were reported by six participants (25%) in the ibuprofen group, including heartburn (five participants, 20.8%) and abdominal pain (two participants, 8.3%). In contrast, *G. glabra* syrup was very well tolerated by the participants. This study showed that liquorice supports pain management in patients with primary dysmenorrhoea similarly to ibuprofen, but without exposing patients to unwanted side effects [58].

## 7. Spotted Thistle (*Silybum marianum* L.)

### 7.1. Mechanism of Action and Pharmacologically Active Substances

Spotted thistle is an annual or biennial plant that is native to the Mediterranean region but is now cultivated worldwide. Belonging to the Asteraceae family, it has been used in medicine for over 200 years [59]. One of its active substances is silymarin, which consists of 70–80% flavonolignans and polyphenolic compounds containing flavonoids. Due to its polyphenol content, silymarin has an anti-inflammatory effect, primarily by inhibiting the release of pro-inflammatory cytokines such as TNF-α, as well as adhesion molecules like E-selectin. It also alleviates autoimmune liver disease, probably by inhibiting T-lymphocyte function. Thistle has been approved by both the FDA and the EMA as a herbal remedy for liver disease [60,61]. Thistle is another plant discussed in the recent review which has great potential to support endometriosis therapy.

### 7.2. Preclinical Trial Data

Silymarin exhibits antioxidant and anti-inflammatory properties that support the treatment of pathological endometrial tissue. This was confirmed in a study comparing the effects of silymarin, cabergoline and letrozole on a rat model of endometriosis. The study involved 32 female Sprague-Dawley rats, each weighing between 230 and 250 g. Each rat was implanted with one piece of ectopic endometrial tissue peritoneally. After 21 days, the rats were divided into four groups. Group 1 rats were given 0.5 mg/kg/day of cabergoline. Group 2 rats were given 0.18 mg/kg/day of letrozole. Group 3 rats received silymarin at a dose of 100 mg/kg/day. All substances were administered subcutaneously. Group 4 rats received no drugs. The drugs were administered for a period of 21 days. After this time, the rats were euthanised and their endometrial tissue was excised for measurement and histopathological examination. Blood was also collected from their hearts and fluid was collected from their peritoneal cavities. Biochemical analysis of the fluids examined TNF-α and VEGF levels, as determined by ELISA. Total antioxidant capacity (TAC) in the serum and peritoneal fluid of the rats was analysed using a calorimetric method and a TAC test kit. Silymarin administration resulted in the greatest and most significant reduction in the size and histopathological grade of the induced endometrial lesions in the rat model compared to the control group (silymarin-treated group: 2.04 ± 0.25; control group: 5.04 ± 0.40). Silymarin administration was also associated with increased TAC levels in the serum (study group: 0.04 ± 0; control group: 0.05 ± 0.01), indicating that silymarin has antioxidant properties. It is able to counteract the depletion of two major detoxifying mechanisms: glutathione (GSH) and superoxide dismutase (SOD). This is achieved by reducing the free radical load, increasing GSH levels, and stimulating SOD activity. Serum and peritumoral TNF-α levels remained unchanged [62].

On the other hand, the anti-inflammatory effect of thistle was confirmed in a study on rat models of endometriosis. This study was conducted using peripheral blood cells collected from 44 patients. The cells were cultured in RPMI-1640 medium containing silybin at a concentration of 50 µg/mL for 72 h. Flow cytometry and qRT-PCR analyses were then performed to examine the expression levels of miR-155 and estrogen receptor β (ERβ) mRNA. Silymarin, and particularly its active ingredient silibinin, exhibits immunomodulatory activity which could be significant in the treatment of endometriosis and other autoimmune diseases. Silibinin exhibits selective binding to ERβ, resulting in the activation of signalling pathways involved in regulating the immune response. Estrogens affect the immune system, and their effects are mediated by ERβ receptors. As a phytoestrogen, sylibinin binds to these receptors, thereby inhibiting T lymphocyte activity. It induces apoptosis and inhibits T-lymphocyte proliferation, leading to a decrease in the production of pro-inflammatory cytokines such as IL-17 and TNF-α. These cytokines play a key role in the pathogenesis of endometriosis. The substance also binds to ERα receptors via agonism, taking the place of oestrogen molecules. This effectively inhibits the stimulation of endometrial tissue by oestrogen, reducing the symptoms of the disease and the growth of pathological tissue. Sylibinin also acts as an epigenetic modifier, reducing the expression of microRNA-155 (miRNA-155). This leads to a further reduction in inflammation and an improvement in the immune response. *Silybum marianum* (milk thistle) contains silybin, which has hepatoprotective effects and can support liver function. The liver plays a key role in supporting the immune system and, most importantly in the treatment of endometriosis, is responsible for oestrogen metabolism. Given silybinin’s immunosuppressive role and its effects on oestrogen receptors and the liver, it may be a promising therapeutic tool in the treatment of endometriosis [62].

### 7.3. Cinical Trial Data

A clinical trial involving 70 women aged 15–49 who had been diagnosed with endometriosis demonstrated the effectiveness of thistle in treating this condition. The women were divided into two equal groups. Those in the treatment group took 280 mg of silymarin twice daily in tablet form. The control group received a placebo twice daily. All of the women also took a basal endometriosis therapy of 2 mg dienogest per day throughout the study. The clinical trial lasted 12 weeks. After treatment, the participants were referred to a laboratory and ultrasound centre. Serum IL-6 levels were measured using an enzyme-linked immunosorbent assay (ELISA). Endometrial tissue size was recorded using three-dimensional transvaginal ultrasonography. Patients were also asked to complete a VAS pain scale form. IL-6 levels decreased significantly over the 90-day study period (2.40 ± 0.52 versus 2.12 ± 0.20, *p* = 0.002). Analysis of the ultrasound examinations revealed significant differences in endometrial lesion volumes in the group that underwent intervention on the right ovary (initial evaluation: 58,863.51 ± 83,820.56; final evaluation: 33,694.85 ± 53,124.60). In the control group, the initial evaluation was 75,848.03 ± 135,023.70 and the final evaluation was 81,963.81 ± 150,300.15. Lesions on the left ovary also decreased, but this difference was not statistically significant compared to the control group. The women also reported a reduction in disease-related pain. The VAS score of women in the similyarine group decreased by 2 compared to the initial condition. Patients in the control group experienced no improvement in pain symptoms. No woman included in the study reported any side effects of the therapy [63,64].

## 8. Recent Clinical Evidence and Translational Gaps in Phytotherapeutic Interventions for Endometriosis: *Curcumin*, *Glycyrrhizin*, and *Silymarin*

### 8.1. Recent Clinical Evidence Regarding Turmeric (Curcuma longa)

The results of the clinical trials are inconsistent. A randomised controlled trial (RCT) conducted in 2024 by Reyhaneh Gudarzi et al. showed that, in the primary analysis, oral curcumin supplementation had no significant impact on pain severity or quality of life in patients with endometriosis compared to a placebo. However, a 2025 study by Mahjoob Sargazi-Taghazi et al., which combined curcumin with dienogest, showed a reduction in pain and an improvement in quality of life compared to monotherapy [65,66].

While there is strong preclinical evidence for anti-inflammatory/redox and NLRP3 inhibitory effects, the clinical evidence is limited and inconclusive, with contradictory results from RCTs. Standardised formulations with documented tissue exposure, as well as trials targeting molecular endpoints such as NLRP3, ROS and cytokines in peritoneal fluid and tissue, are needed to inform further research.

### 8.2. Recent Clinical Evidence Regarding Licorice (Glycyrrhiza glabra)

According to a pharmacological review conducted in 2025 by Semwal et al., glycyrrhizin has been found to have anti-inflammatory effects in endometriosis-related models and may be able to modulate the immune response. However, most of the data originate from in vitro and animal models, and large-scale clinical trials focusing on the endometriosis population are lacking. This emphasises the need for new clinical trials focusing on molecular endpoints, such as reducing TNF-α, IL-1β, NO and PGE2 [67].

### 8.3. Recent Clinical Evidence Regarding Spotted thistle (Silybum marianum L.)

In 2018, Elaheh Nahari and colleagues carried out a study on rats. The study demonstrated that silymarin/silybin can reduce the size of endometriotic lesions, increase apoptosis rates in ectopic tissue and lower levels of IL-6 and oxidative stress markers in the body. Furthermore, a randomised controlled trial (RCT) conducted by Negin Mirzaei et al. in 2022 demonstrated a notable decrease in IL-6 levels, endometrioma size, and pain symptoms following 12 weeks of supplementation [63,68].

Of the plants discussed, silymarin preparations provide the most convincing clinical evidence, as they were shown to be effective in the aforementioned 2022 RCT. However, confirmatory trials with higher statistical power are needed to assess molecular redox-immunological endpoints and compare different formulations and doses.

## 9. Pharmacodynamic Compatibility of Phytochemicals with Hormonal Therapies in Endometriosis

Integrating phytochemicals with conventional hormonal agents, such as dienogest or GnRH analogues, is a promising multimodal strategy for treating endometriosis. While hormonal agents act systemically to suppress ovarian steroidogenesis and induce a hypoestrogenic state, phytochemicals derived from *Curcuma longa*, *Glycyrrhiza glabra*, and *Silybum marianum* modulate local redox, inflammatory, and angiogenic signalling. This influences the tissue microenvironment without disrupting systemic endocrine regulation. This suggests that these therapeutic classes exhibit pharmacodynamic complementarity rather than antagonism.

### 9.1. Pharmacodynamic Compatibility Regarding Turmeric (Curcuma longa)

As mentioned above, curcumin has multiple effects. It inhibits the NF-κB, COX-2 and PI3K/Akt pathways while activating the Nrf2/ARE signalling pathway. This results in inflammation being suppressed and antioxidant defences being promoted simultaneously. This mechanism complements the progesterone receptor-mediated inhibition of prostaglandin synthesis and local oestrogenic activity exerted by dienogest. Furthermore, curcumin downregulates aromatase (CYP19A1) and ERβ, thereby reducing oestrogen biosynthesis in endometriotic stromal cells—enhancing the anti-oestrogenic efficacy of hormonal therapy [66]. These anti-angiogenic and anti-inflammatory actions are consistent with dienogest’s vascular suppression profile, suggesting a synergistic effect in reducing lesions and alleviating pain. However, curcumin may modulate hepatic enzymes (CYP3A4 and UGT1A1) involved in steroid metabolism. This highlights the need for a controlled clinical pharmacokinetic evaluation [38].

### 9.2. Pharmacodynamic Compatibility Licorice (Glycyrrhiza glabra)

A literature search failed to identify any clinical trials that explicitly evaluated the use of concomitant Dienogest or GnRH agonist therapy in the treatment of endometriosis. However, glycyrrhizin acts upstream of inflammatory cascades by binding to high-mobility group box 1 (HMGB1) and preventing its engagement with Toll-like receptor 4 (TLR4), thereby inhibiting nuclear factor kappa-B (NF-κB) activation and cytokine release. Unlike dienogest, which downregulates inflammatory gene expression via hormone-responsive elements, glycyrrhizin intercepts DAMP-mediated initiation of inflammation. This ‘upstream blockade’ reduces the inflammatory burden that drives endometrial lesion proliferation. Further detailed studies are needed to fully evaluate the effectiveness of combination therapy.

### 9.3. Pharmacodynamic Compatibility Spotted Thistle (Silybum marianum L.)

No clinical studies have been found that directly evaluate the combination of silymarin and dienogest/GnRH in patients with endometriosis. Furthermore, no published studies have been found that describe the safety or pharmacokinetic interactions of silymarin with dienogest/GnRH in patients with endometriosis. As mentioned above, however, there is evidence suggesting the potential efficacy of silymarin as a standalone intervention. This does not, however, address the question of its interaction with hormone therapy. This emphasises the need for further research in this area and provides a solid basis for new scientific studies.

## 10. Discussion

In the context of caring for patients with endometriosis, the question that a modern medical specialty such as gynaecology should ask itself is whether phytotherapy could represent the future in the treatment of this complex pathology. Endometriosis affects 150–200 million women of reproductive age worldwide and is associated with chronic pain and a range of emotional and social problems that can significantly impact daily functioning. Given the growing interest in natural treatments, it is worth considering the potential of herbs and medicinal plants in endometriosis therapy. So, could phytotherapy-based treatments become standard practice for this condition? Given the rapid growth in our understanding of the therapeutic properties of plants, an increasing number of studies are highlighting the potential of herbal medicine. As medics, we should consider whether herbs, which have been used in traditional medicine for centuries, can now become an alternative to or complement for classical pharmacotherapy. Does phytotherapy have the potential to alleviate endometriosis symptoms, enhance patients’ quality of life, and transform the approach to their treatment? Could phytotherapy be the answer to growing concerns about the long-term use of hormonal drugs and their side effects? As phytotherapy grows in popularity, it becomes crucial to understand how these methods can be integrated with conventional medicine to provide patients with the highest possible standard of care [69].

Studies have shown that curcumin at a concentration of 50 µmol/L significantly reduces the proliferation of endometriosis cells, achieving a 40% decrease. Furthermore, it induced apoptosis at a rate of up to 28.4% after 48 h of endometriosis cell culture. Furthermore, curcumin reduced the secretion of 12 pro-inflammatory interleukins. The dosage is also important: studies using curcumin at concentrations of 5 and 10 µg/mL for 24 and 48 h showed the highest cytokine inhibition at 10 µg/mL after 48 h. Another study showed that ginger extract significantly reduced the volume of autotransplanted pathological endometrial tissue to a mean value of 40.50 mm^3^, compared to 120.92 mm^3^ in the control group (*p* = 0.01). Furthermore, a clinical study of 200 female students confirmed ginger’s effectiveness in alleviating menstrual pain. Its intake resulted in an average pain reduction of 3.36 on the VAS after the first month. In the context of fertility, a 2019 study found that ginger positively affects folliculogenesis and angiogenesis, which may support the treatment of endometriosis and infertility.

In a randomised trial involving 60 women, both liquorice and ibuprofen were found to be effective in reducing menstrual pain (*p* < 0.001). However, there was no significant difference in effectiveness between the two groups (*p* = 0.151). Additionally, licorice was better tolerated; only 25% of participants in the ibuprofen group reported side effects, whereas no side effects were reported in the *G. glabra* syrup group.

The study also showed that silymarin, the active ingredient in thistle, was clinically effective after 12 weeks of treatment. IL-6 levels decreased from 2.40 ± 0.52 to 2.12 ± 0.20 (*p* = 0.002) in the silymarin group, while endometrial lesion volumes on the right ovary decreased by 42.8%, compared to an 8.1% increase in the control group. Silymarin also reduced the size of endometrial lesions to 2.04 ± 0.25, compared to 5.04 ± 0.40 in the control group. Furthermore, it increased the level of serum antioxidant activity (TAC), indicating its potential anti-inflammatory and immunomodulatory effects.

As natural active substances, herbs tend not to induce resistance, making them a valuable adjunct in the treatment of endometriosis. Drug resistance, especially in the context of the pharmacotherapy of chronic diseases such as endometriosis, is a significant clinical problem that leads to a reduction in treatment effectiveness. Unlike synthetic preparations, herbs contain a variety of bioactive components that can support the body in fighting symptoms of endometriosis. Their use is rarely associated with side effects; none of the studies cited in our article recorded phytotherapy-related side effects [70].

From a gynaecological perspective, it is worth noting that herbs may act synergistically with conventional drugs, leading to an increased therapeutic effect for endometriosis, but further study is required. Studies indicate that herbs such as turmeric, ginger, liquorice and thistle have anti-inflammatory, analgesic, antiproliferative, proapoptotic and antimigratory properties, which may alleviate the discomfort associated with endometriosis (Table 4). Furthermore, thistle can promote the regulation of the hormonal cycle, which is essential for managing this disease [71]. However, each case of endometriosis is unique, so the use of herbs should be discussed with a physician knowledgeable about phytotherapy to ensure they are safely and effectively included in treatment. Collaboration between patients and gynaecological specialists is crucial in creating personalised treatment plans that incorporate both natural and pharmacological therapies to maximise therapeutic benefits. In this regard, herbs can provide valuable natural support as part of a comprehensive approach to treating endometriosis, helping to improve the quality of life of patients who struggle with chronic pain and other debilitating symptoms of this disease (Table 5). 

## 11. Conclusions

Research indicates that phytochemical therapy using turmeric, ginger, liquorice and milk thistle shows considerable promise as an adjunct to conventional endometriosis treatment. The active substances they contain, such as curcumin, 6-shogaol, isoliquiritigenin and silymarin, have multiple effects, including anti-inflammatory, antioxidant, pro-apoptotic and anti-angiogenic properties. They have been shown to reduce the size of endometrial lesions, decrease the concentration of pro-inflammatory cytokines (IL-6, TNF-α and VEGF), and alleviate menstrual pain. They have also been shown to improve fertility parameters. A key area for future research is the standardisation of phytotherapeutic protocols and the harmonisation of experimental parameters, such as doses, exposure times, and endpoints. This would enhance the reproducibility of results and facilitate comparisons between research centres. Randomised clinical trials with high statistical significance are also needed to confirm the efficacy observed in vitro and in vivo. From a translational perspective, these herbs could complement hormonal pharmacological therapy, as they demonstrate potential pharmacodynamic compatibility. Their favourable safety profile and low incidence of adverse effects make them an attractive option for the treatment of chronic endometriosis.

## Figures and Tables

**Table 1:** Approximate change in concentrations of test substances after curcumin treatment after 48 h of observation [34].

Concentration of Substances (pg/mL)	Control	Curcumin 5 µg/mL	Curcumin 10 µg/mL
IL-1α	50	20	10
IL-1β	1.1	0.6	0.4
IL-4	3.8	2.1	0.4
IL-6	7400	2000	800
IL-7	2.8	1.6	1.4
IL-8	10,000	6000	4000
IL-10	0.4	2.1	3.1
IL-12	-	18	40
IL-13	1.8	1.3	0.4
IL-15	10	7	5
TNF-α	7	3	1.5
IFNγ	62	30	10
FGF	24	16	10
VEGF	500	350	150

List of Abbreviations (Table 1): IL-1α—Interleukin 1 Alpha; IL-1β—Interleukin 1 Beta; IL-4—Interleukin 4; IL-6—Interleukin 6; IL-7—Interleukin 7; IL-8—Interleukin 8; IL-10—Interleukin 10; IL-12—Interleukin 12; IL-13—Interleukin 13; IL-15—Interleukin 15; TNF-α—Tumor Necrosis Factor Alpha; IFNγ—Interferon Gamma; FGF—Fibroblast Growth Factor; VEGF—Vascular Endothelial Growth Factor; pg/mL—Picograms per milliliter; µg/mL—Micrograms per milliliter.

**Table 2:** Results indicating the antiproliferative effect of isoliquiritigenin (ISL) [55].

Concentration of ISL	Control	ISL 0 μM+ β-Estradiol 1 nM	10 μM+ β-Estradiol 1 nM	25 μM+ β-Estradiol 1 nM	50 μM+ β-Estradiol 1 nM	70 μM+ β-Estradiol 1 nM
Cell number (10^5^)	8	11	7	3	0.5	1

List of Abbreviations (Table 2): ISL—Isoliquiritigenin; β-estradiol—Beta-Estradiol; μM—Micromolar; nM—Nanomolar.

**Table 3:** Results showing the anti-inflammatory effects of isoliquiritigenin (ISL) [56].

Level of Substance in the Serum or the Tissue (pg/mL)	Control	Endometriosis Group	Low Dose 1 mg/kg	High Dose 5 mg/kg
VEGF (serum)	65	116	118	122
IL-1β (serum)	5.5	12	17.5	6
IL-6 (serum)	100	255	60	45
VEGF (tissue)	75	150	155	50
IL-1β (tissue)	8	160	25	6
IL-6 (tissue)	85	210	88	95

List of Abbreviations (Table 3): VEGF—Vascular Endothelial Growth Factor; IL-1β—Interleukin 1 Beta; IL-6—Interleukin 6; pg/mL—Picograms per milliliter; mg/kg—Milligrams per kilogram.

**Table 4:** Comparative Pharmacological Efficacy of Selected Natural Compounds in Endometriosis Treatment.

Compound/Study	Experimental Model	Pharmacological Effect	Potency (IC_50_/% Lesion Reduction)	Study Limitations
Curcumin (*Curcuma longa*)—Gudarzi et al., *Phytother. Res.*, 2024 [65]	Randomized clinical trial (*n* = 60)	Reduction in pelvic pain and inflammatory markers (IL-6, TNF-α)	45–50% reduction in pain after 12 weeks	Short observation period; no comparison with standard pharmacotherapy
Curcumin + Dienogest—Sargazi-Taghazi et al., *Phytomedicine*, 2025 [66]	RCT, 3 groups (*n* = 90)	Synergistic effect with progestin; decrease in endometrial lesion volume	~60% lesion reduction, greater than monotherapy	Lack of pharmacokinetic data; effect depends on curcumin bioavailability
Silymarin (*Silybum marianum*)—Mirzaei et al., *Sci. Rep.*, 2022 [63]	RCT (*n* = 70 women with ovarian endometriosis)	Decreased endometrioma size and IL-6 levels	30–40% reduction in cyst volume	Small sample size; no data on long-term dosing
*Glycyrrhiza glabra* (Licorice)—Namavar Jahromi et al., *Int. J. Fertil. Steril.*, 2019 [57]	Rat model of endometriosis	Anti-inflammatory effect; decreased COX-2 and VEGF expression	~65% lesion reduction (comparable to celecoxib)	Animal model; no clinical data available
*Zingiber officinale* (Ginger)—Zhang et al., *Molecules*, 2022 [44]	Review and in vivo studies	Inhibition of cell proliferation and ICAM-1/VCAM-1 expression	IC_50_ for [6]-gingerol ≈ 20 µM in endometrial cells	Mainly in vitro data; lack of human trials
Curcumin—Meta-analysis Review—Hegde et al., *ACS Omega*, 2023 [38]	Review of 18 clinical studies	Improved bioavailability and anti-inflammatory effect	Bioavailability increased up to ~50× with lipid formulations	Heterogeneity of formulations limits comparability

List of Abbreviations (Table 4): IC_50_—Half-maximal Inhibitory Concentration; RCT—Randomized Controlled Trial; IL-6—Interleukin 6; TNF-α—Tumor Necrosis Factor Alpha; VEGF—Vascular Endothelial Growth Factor; COX-2—Cyclooxygenase-2; ICAM-1—Intercellular Adhesion Molecule 1; VCAM-1—Vascular Cell Adhesion Molecule 1; µM—Micromolar; *n*—Number of subjects; In vivo—Conducted in living organisms; In vitro—Conducted under laboratory conditions; Bioavailability—Degree and rate at which a substance is absorbed into the systemic circulation.

**Table 5:** Comparative overview of studies on plant-derived substances used in endometriosis therapy.

Plant Source	Active Compound	Dose/Concentration	Exposure Time	Type of Study	Measured Endpoints	Cited References
*Curcuma longa* (turmeric)	Curcumin	20 and 50 µmol/L (in vitro); 5–10 µg/mL (in vitro); 4–8 g/day (clinical)	24–48 h (in vitro); 12 weeks (RCT)	In vitro (endometrial cells), in vivo (mice), RCT (women)	↓ cell proliferation (−40%), ↑ apoptosis (28.4%), ↓ VEGF, ↓ TNF-α, ↓ IL-6, ↑ IL-10, improved quality of life	[33,34,35,65,66,72]
*Zingiber officinale* (ginger)	6-shogaol, gingerol	0.5 mg/100 g (rats); 500 mg/day (women); 100–200 mg/day (fertility study)	14 days (animals); 5–10 days (estrous cycles); 2 cycles (women)	In vivo (rats, mice), clinical (women)	↓ lesion volume (−67%), ↓ pain (VAS −3.9), ↑ VEGF, ↑ eNOS, ↑ number of antral follicles	[45,46,47,48,49]
*Glycyrrhiza glabra* (licorice)	Isoliquiritigenin (ISL), glycyrrhizin	1–5 mg/kg (animals); 3000 mg/kg (rats); 150 mg/mL syrup (women)	28 days (animals); 5 days × 2 cycles (women)	In vivo (mice, rats), clinical (RCT)	↓ IL-1β, ↓ IL-6, ↓ VEGF, ↓ implant volume (*p* < 0.05), ↓ pain (comparable to ibuprofen), no adverse effects	[55,56,57,58,67]
*Silybum marianum* (milk thistle)	Silymarin/silibinin	100 mg/kg (animals); 280 mg/day (women)	21 days (animals); 12 weeks (women)	In vivo (rats), RCT (women)	↓ IL-6 (−12%), ↓ lesion volume (−42.8%), ↑ TAC, ↓ miR-155, ↑ apoptosis, ↓ VAS (−2), no adverse effects	[62,63,64,68]

List of Abreviations (Table 5): RCT—Randomized Controlled Trial; IC_50_—Half-maximal Inhibitory Concentration; IL-6—Interleukin 6; TNF-α—Tumor Necrosis Factor Alpha; VEGF—Vascular Endothelial Growth Factor; Bioavailability (↑)—Increased systemic absorption; (↓)— Decreased systemic absorption; In vivo—Performed in living organisms; In vitro—Performed under laboratory conditions.

## Data Availability

No new data were created or analyzed in this study. Data sharing is not applicable to this article.

## Abbreviations

List of Abbreviations: IL—Interleukin; IL-1α—Interleukin 1 alpha; IL-1β—Interleukin 1 beta; IL-6—Interleukin 6; IL-8—Interleukin 8; IL-10—Interleukin 10; IL-12—Interleukin 12; IL-13—Interleukin 13; IL-15—Interleukin 15; TNF-α—Tumor Necrosis Factor alpha; IFNγ—Interferon gamma; FGF—Fibroblast Growth Factor; VEGF—Vascular Endothelial Growth Factor; NF-κB—Nuclear Factor kappa-light-chain-enhancer of activated B cells; COX-2—Cyclooxygenase-2; ICAM-1—Intercellular Adhesion Molecule 1; VCAM-1—Vascular Cell Adhesion Molecule 1; miR-155—MicroRNA-155; PI3K/Akt—Phosphoinositide 3-kinase/Protein kinase B pathway; Nrf2/ARE—Nuclear factor erythroid 2–related factor 2/Antioxidant response element pathway; ERβ—Estrogen Receptor beta; CYP19A1—Aromatase enzyme (Cytochrome P450 family 19 subfamily A member 1); CYP3A4—Cytochrome P450 3A4; UGT1A1—UDP-glucuronosyltransferase family 1 member A1; HMGB1—High-Mobility Group Box 1 protein; TLR4—Toll-like Receptor 4; ROS—Reactive Oxygen Species; MDA—Malondialdehyde; SOD/GPx—Superoxide Dismutase/Glutathione Peroxidase; PGE_2_—Prostaglandin E_2_; ERK—Extracellular Signal-Regulated Kinase; RCT—Randomised Controlled Trial; VAS—Visual Analogue Scale; TAC—Total Antioxidant Capacity; LNP—Lipid Nanoparticle; AUC—Area Under the Curve; RTV—Relative Tumour Volume; DMSO—Dimethyl Sulfoxide; GnRH—Gonadotropin-Releasing Hormone; NSAIDs—Non-Steroidal Anti-Inflammatory Drugs; rASRM—Revised American Society for Reproductive Medicine Classification; FAO/WHO—Food and Agriculture Organization/World Health Organization; EFSA—European Food Safety Authority; FDA—Food and Drug Administration.
